# Integrating a 19F MRI Tracer Agent into the Clinical Scale Manufacturing of a T-Cell Immunotherapy

**DOI:** 10.1155/2017/9548478

**Published:** 2017-11-02

**Authors:** Charles F. O'Hanlon, Tamara Fedczyna, Shannon Eaker, William D. Shingleton, Brooke M. Helfer

**Affiliations:** ^1^Celsense Inc., 603 Stanwix Street Suite 390, Pittsburgh, PA 15222, USA; ^2^GE Healthcare, 100 Results Way, Marlborough, MA 01752, USA; ^3^GE Healthcare, The Grove Center, White Lion Road, Amersham, Buckinghamshire HP7 9LL, UK

## Abstract

Leukocyte immunotherapies have made great progress in the treatment of cancer. Recent reports on the treatment of B-cell malignancies using Chimeric Antigen Receptor and affinity enhanced T-Cell Receptor therapies have demonstrated encouraging clinical results. As investigators begin to explore the treatment of solid tumors with these cells, the hurdle of evaluating T-cell homing to and persistence at the site of disease remain. Significant challenges regarding the GMP manufacture and administration of a therapeutic dose of millions to billions of transduced T-cells remain. Here we report on the application of a clinically authorized 19F MRI tracer agent to human T-cells, employing state-of-the-art methods and equipment in the manufacture of a cellular therapy. Using a general T-cell expansion protocol and clinical scale industrial bioreactors, we show 19F labeling without detriment to the product +/− cryopreservation. While the incorporation of the 19F tracer is not trivial, it is just one of the many steps that can aid in progression of a therapeutic to and though the clinic. Combining the MRI tracking capabilities, safety profiles, and clinical sensitivity of this method, this application demonstrates the ability of 19F MRI to be used in industrial scale applications to visualize the spatial fate of cellular therapeutics.

## 1. Introduction

In the past 5 years, the successful treatment of cancer patients with autologous T-cells that have been engineered to recognize and kill tumors has been reported in the academic literature [1–8]. Although the total number of patients treated is relatively small, the unexpectedly high rates of complete response have provoked global reaction by other academic investigators, large pharmaceutical companies, and financial markets to allocate significant resources to translate these therapies into commercial products that can be economically delivered to patients.

The enabling breakthrough for these new therapies is the targeting of tumor cells with aberrant patterns of gene expression versus the originating tissue type [9]. These therapies are typically referred to as CAR T-cells (Chimeric Antigen Receptor-T-cells) or TCR cells (T-Cell Receptor cells) but may involve NK cells, mixed lymphocytes, and T-cells and NK cells derived from allogenic cell lines. In this study, we refer to them generically as engineered T-cell therapies. The engineered aspect of these cells typically involves genetically modifying immune cells in culture to express a receptor with an affinity for a target expressed by the tumor cells [10].

A second engineering aspect is large scale processes to genetically modify tens of millions of cells in culture, extracting the “unused” proteins and virus used in the transduction process, expanding the population of modified cells, and testing the cell product to verify that it meets predetermined release criteria. Integrating manufacturing of complex proteins and virus particles, traditional tissue culture, large scale transduction, and quality assurance on an industrial scale in a patient-centric process is a daunting undertaking that is currently being developed in real-time on a global basis [10, 11]. This process involves many empirical steps: testing a particular technology on a stand-alone basis and retesting the technology integrated in the putative large scale process.

The rich market valuations for companies specializing in engineered T-cell therapies indicate that investors expect therapeutic developers to rapidly overcome the many manufacturing challenges for these treatments and advance beyond the treatment of blood cancers to solid tumors. Of the 1.7 million new cases of cancer in the US each year, less than 1% involve Acute Lymphoblastic Leukemia (ALL), the most successful treatment to date, and less than 10% involve leukemia, lymphoma, and myeloma [12].

Investigators and developers of engineered T-cell therapies are currently considering a large number of receptors and targets to treat solid tumors. Unfortunately, there is no high throughput* in vitro* method or data demonstrating the relevancy of preclinical models involving xenograft human tumors and engineered human immune cells. Like the work in ALL, the path forward will involve multiple early clinical studies. To accelerate the process, developers must embrace new technologies that answer fundamental questions regarding migration, persistence, and tumor killing for new receptor and target combinations. Definitive pharmacokinetic (PK) data for engineered T-cells will rapidly identify promising therapies and may provide further guidance for dosing strategies, different routes of administration, the ablation of endogenous immune cells, and the simultaneous administration of check point inhibitors or other immunotherapies.

The ideal cellular imaging technology for the development of PK data meets several criteria including being a quantitative, sensitive, biocompatible (viability preserving and nongenetically modifying), long lived, and nondilutive [13]. Most imaging technologies meet some but not all of these characteristics, with varying pros and cons of each modality, requiring a balance of wants and needs in examining cellular migration and persistence. Common imaging modalities for clinical cell tracking include MRI, PET, and SPECT. Ultrasound has also been used but is less common (imaging modalities reviewed extensively elsewhere, [13–17]). Here we will examine a nonradioactive perfluorocarbon based imaging agent that has been used clinically and is able to be visualized and quantified by fluorine MRI [18, 19].

Incorporation of fluorine labeling has been well demonstrated in the literature [18–24] but only on a small scale, cultures involving small volume flasks and not liters of media. In this paper we describe one of the empirical steps in the process of evaluating a new therapeutic component, namely, cell labeling. Here we report on a set of experiments that demonstrate that Cell Sense (CS-1000), a 19F MRI cell tracking agent used to develop clinical pharmacokinetic data for cell therapies, can be integrated into an industrial process for manufacturing an engineered T-cell therapy.

## 2. Methods

### 2.1. T-Cell Generation

Lymphocytes were isolated from buffy coats (Central Blood Bank, Pittsburgh PA) by Ficoll (GE Healthcare) and gradient separation (Percoll, GE Healthcare) as described previously [25]. Lymphocytes were stimulated with 2.5 ug/mL PHA (Roche) and cultured in Iscove's Modified Dulbecco's Medium (IMDM, Gibco) containing 10% human AB serum (Sigma), 200 IU/mL IL-2 (GE Healthcare), 0.5% Penicillin, Streptomycin, Glutamine mix (PSG, Gibco), and 600 mg/L glucose (Gibco).

### 2.2. Cellular Expansion

Cells were expanded in 2 L Perfusion Cellbags in Wave 2/10 (Xuri W5) bioreactors with perfusion capability (GE Healthcare). Cells were initially seeded at 2.5–5 × 10^5^ cells/mL with 5% CO2 and 6 RPM (rock per minute). Culture volume was increased, keeping cells at 5 × 10^5^ cells/mL until 1 L of media is reached. Once the culture volume was above 750 mL, rock was increased to 8 RPM. Perfusion was started once culture volume reaches 1 L and the rock rate was increased to 10 RPM. Days 1-2 were set for 250 mL of perfusion. Days 3–5 were set at 500 mL. Cultures were grown for 7 days reaching up to 2 × 10^6^ cells/mL.

### 2.3. 19F Labeling of Human T-Cells

Cell Sense (CS-1000 ATM, Celsense Inc.) was infused via a vented vial spike (ICU Medical) into the bioreactor. Reagent was either pumped in via the bioreactor pump or fed by gravity. Vials are filled with 27 mL of reagent, with the spike 25 mL being delivered per vial. Cells were labeled at 10 mg/mL CS-1000 ATM for the final 24 hours of culture.

### 2.4. Cellular Viability

Cellular viability was determined by Trypan Blue exclusion.

### 2.5. Cellular Phenotype

Cell surface immunostaining analysis was performed using a BD FACS Caliber flow cytometer. Fluorescein isothiocyanate (FITC) CD3 and CD4 (Beckman Coulter Immunotech) and phycoerythrin (PE) CD8 (Beckman Coulter Immunotech) and CD32 (Biolegend) conjugated antibodies were used with their associated control antibodies (Beckman Coulter Immunotech and Biolegend) to assess the cellular population. Before staining, cells were washed in phosphate buffered saline (PBS) and blocked in 20% human AB serum for 10 minutes. Samples were then incubated with fluorescent antibodies at suggested concentrations for 20 minutes. After washing with PBS, samples were analyzed. Data analysis was performed using BD CellQuest Pro Software.

### 2.6. Nuclear Magnetic Resonance (NMR) Analysis of Labeled Cells

To assay the fluorine content of the cells after labeling, cell pellets of a known number of cells (≥3 × 10^6^ cells) were lysed with 1% Triton-X 100 (Sigma Aldrich) and a fluorine reference solution (trifluoroacetic acid, TFA) for a final concentration of 0.05% TFA. The lysed solution was placed in a 5 mm quartz NMR tube and read using a Bruker AVANCE spectrometer (Bruker, Billerica MA) operating at 282 MHz. 19F NMR spectra with both PFPE and TFA peaks were obtained and the ratios of the integrated areas under the peaks were used to calculate the mean 19*F*/cell or *F*/*c* as described previously [24, 26].

### 2.7. Determination of Labeled Population

Cells were labeled with Cell Sense CS-ATM DM Green (CS-DM), a preclinical grade, fluorescent green conjugated version of CS-1000 ATM as performed above. Cells were washed of excess reagent and label uptake assessed by flow cytometry. Percentage of labeled population was determined by comparing the histograms for the 488 emission channel between unlabeled and CS-DM labeled cells.

### 2.8. Cryopreservation

Cells were pelleted and resuspended in 20% serum (Sigma Aldrich) 10% dimethyl sulfoxide (DMSO, brand) containing media which is slowly brought down to −80°C before transferring to liquid nitrogen.

## 3. Results

### 3.1. The Logistics of Labeling at Industrial Scale

**Figure 1:**
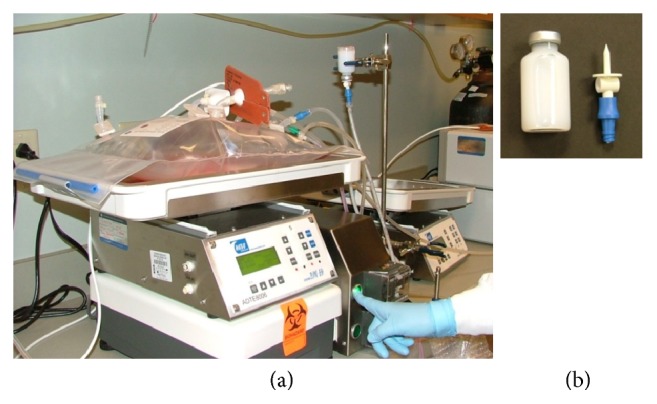
*Incorporation of cellular labeling.* Human T-cells were expanded to 1 L of culture in a GE Wave 2/10 bioreactor. (a) By adding CS-1000 ATM through a vented spike, a closed sterile system was able to be maintained. Vials containing 27 mL of CS-1000 ATM were fed either by gravity (not shown) or by peristaltic pump through a vented vial spike. With this method of delivery, 25 mL of reagent is delivered per vial. (b) 27 mL vial imaged with the vented spike.

Introducing an imaging agent into clinical preparations requires the properties of the therapeutic and the manufacturing protocol for cellular preparation be unaltered by its addition. Preclinical studies have shown the efficacy of the reagent at experimental levels and in the clinic at cellular doses of 10^6^ cells [18, 20–24]. In the area of T-cell therapies, higher doses of cells and large volumes are often required for cellular expansion. Scale, in this context, takes into consideration billions of cells and its relationship to the volume of label, along with the ability to maintain sterility within the system. These parameters at a small scale were not an obstacle when compared to larger clinical scale preparations. The issue of scale was addressed by taking product packaging volume from 4 mL to 27 mL. The 27 mL packaging resulted in 25 mL of delivered reagent with 2 mL of hold-up volume when administered by vented spike (Figure 1). Small scale preparations involved the addition of reagent to a flask in a tissue culture hood, while large scale preparations are often performed in a clean room, where a closed system is preferred. The addition of the larger packaging size and vented spike delivery allows for larger volume administration without the transfer of the bioreactor to a culture hood. Using this method, reagent can be pumped in via the bioreactors peristaltic pump or simply gravity fed. Adding to the adaptability of the process, cellular labeling was performed in the final 24 hours of cellular expansion as not to increase the time or complexity of product preparation.

### 3.2. Uptake and Viability

**Figure 2:**
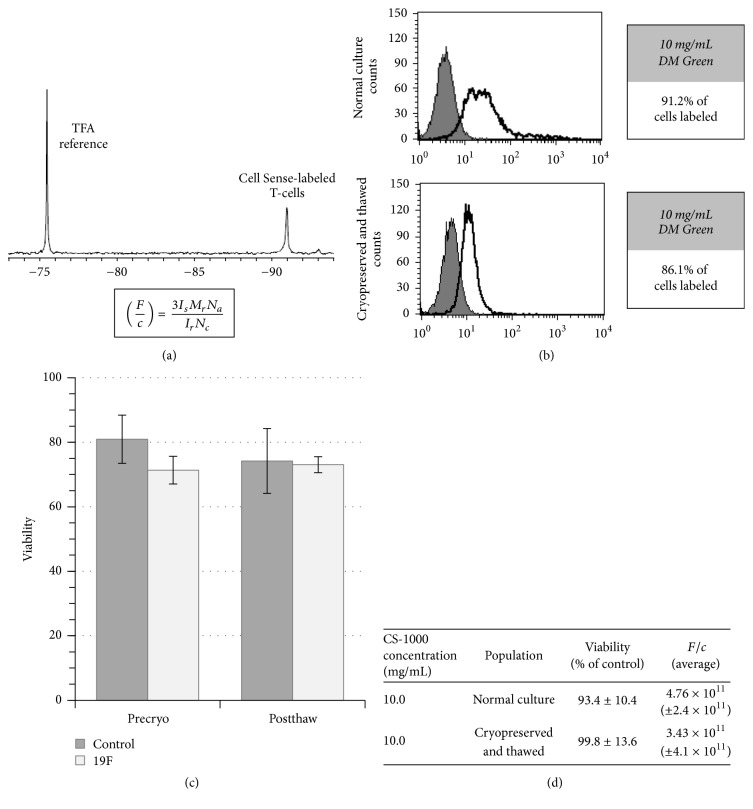
*Label uptake and cellular viability after label.* (a) CS-1000 ATM produces a single major spectral peak when detected by NMR. Using the integration values of the labeled cells and TFA reference, the 19F atoms/cell (*F*/*c*) can be calculated. Is = integrated area of major peak of the cell pellet, Mr = moles of TFA reference (three 19F per TFA molecule already reflected in equation), Na = Avogadro's number, Ir = integrated area under TFA reference peak, and Nc = number of cells in pellet. (b) Labeling with a green fluorescently conjugated version of the reagent, cellular uptake of the reagent was examined by flow cytometry (n2). (c) Comparison of the viability of labeled and unlabeled cells both before and after cryopreservation (precryo n5, postthaw n2). (d) Table summarizing the viability and label uptake of groups of cells.

Cellular viability is a common release criterion for therapeutic cells. Keeping the requirements for an imaging reagent in mind, cells were examined under two categories: normal healthy donor expanded T-cells and cryopreserved T-cells. The two categories of cells were used to examine clinical scale labeling and expansion as well as the storage of labeled cells to assess labeling properties for stored and/or shipped cells. Prior studies showed that T-cells are labeled effectively at 10 mg/mL, which is the constant dose used throughout the studies (data not shown). Cells were labeled at 10 mg/mL in 1 L of media for 24 hours. The label possesses a single major spectral peak by NMR, which when compared to a trifluoroacetic acid standard (TFA) allows for the calculation of *F*/*c* (19*F*/cell, Figure 2(a)). All cell groups demonstrated labeling of 10^11^ 19*F*/cell (*F*/*c*). Using Trypan Blue exclusion, we clearly demonstrated that cellular viability was maintained (Figures 2(c) and 2(d)). Importantly, cryopreserved cells maintain cellular health and retain label after thaw (Figure 2(c)). Viability and *F*/*c* are summarized for each category of cells in Figure 2(d). Examining the degree of label retention in nonengineered cells, a fluorescently conjugated form of Cell Sense was used to show the distribution of cellular label (Figure 2(b)). Flow cytometry confirmed that ~90% of the T-cell preparation was labeled with reagent.

### 3.3. Cellular Characterization

**Figure 3:**
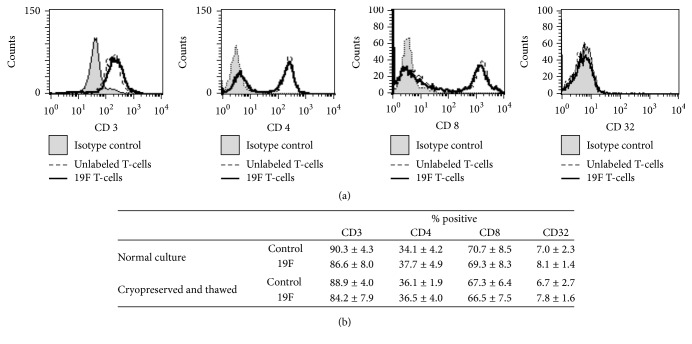
*Phenotypic examination of labeled cells*. Labeled and unlabeled T-cell populations are compared for the presence of the following phenotypic markers: CD3, CD4, CD8, and CD32. (a) Histograms comparing cellular populations labeled with conjugated antibodies for the depicted surface marker or isotype control. (b) Table summarizing the cellular populations (n2) presenting +/− CS-1000 ATM labeling and +/− cryopreservation.

A cellular therapeutic requires a clear definition and characterization of the cellular product. Before therapeutics is released for patient administration, a number of release criteria are assessed. A panel of surface markers was used to assess labeled and unlabeled cells in each of the previously mentioned categories (recently expanded and cryopreserved). Different immunotherapeutic T-cells have different release criteria; in this study we examined the cellular populations for the CD3 T-cell maker, CD8 cytotoxic T-cell marker, CD4 T helper cell marker, and CD32 myeloid and B-cell maker. The ratios of CD8 and CD4 cells were maintained with a limited number of CD32 (nonlymphocyte) cells present (Figure 3). Importantly, these ratios were maintained after cryopreservation. The composition of engineered cells will vary by the way in which they are generated, with the desired ratios of CD4 : CD8 cell varying by manufacture; based on the labeling performed here, we would not anticipate the ratios to change in other expansion protocols.

## 4. Discussion

Early Phase 1 trials primarily look for evidence of drug safety, but pharmacologic activity and early indications of efficacy are often explored. Early Phase 1 trials typically involve escalating dosage and a single route of administration. In consideration of early Phase 1 trials, the FDA states in their guidance for Cellular and Gene Therapy Products that evidence of the cell therapy products persistence and activity should be monitored. Further, the guidance document suggests monitoring the point of administration and site of intended activity, as well as other potential migratory sites or abnormal cell behavior [27]. Pharmacokinetic (PK) data for a cell therapy product* in vivo* would help answer some of these unknowns. The cell therapy results to date are promising [1–8]; however cellular therapies face a number of challenges before coming of a widely available treatment option. Manufacturing scale-up and obtaining pharmacokinetic data are two such challenges to be overcome, both obstacles considered throughout this manuscript.

Incorporation of an imaging agent into a clinical protocol requires that the imaging agent does not alter the cellular therapy, either in health or in function, and is capable of being imaged clinically [13]. Earlier experiments demonstrate the labels ability to maintain cellular health and function in multiple cell types [18, 20–24] and to be imaged at clinically relevant scan times [19]. For ease of use, having the label be easily incorporated into cellular processing was also important. CS-1000 ATM does not require the addition of transfection agents, which eases the incorporation process. Our studies demonstrate an additional simplification; by increasing the packaging and dose of deliverable reagent per vial, we were able to maintain the ease of use for large clinical scale manufacturing (Figure 1). A degree of the design of expansion protocols involve expansion of the culture in bioreactors in a clean room environment, factoring in the attachments (tubing) feeding into the bioreactors for the continued culture. Having a 25 mL dose of cellular label being administered to existing lines of a bioreactor via a vented spike eliminates the transfer of the culture to a biologic safety cabinet and lends itself to the industrial manufacturing process. Through the consideration of these scaling components, CS-1000 ATM was able to overcome previously undemonstrated questions of labeling cells at clinical scale.

The addition of the 19F imaging agent to the manufacturing process did not alter common release criteria, namely, the cellular health or phenotype of the culture (Figures 2 and 3, resp.). An important aspect of adding an internalized label for cellular trafficking is not to alter these criteria. Cells were labeled with an average of 3.7 × 10^11^ *F*/*c*. Cryopreservation of samples was also examined. When cells are being tested for release criteria, shipped, or stored for repeat dosing, cryopreservation may be necessary. Cellular phenotype, health, and cellular label were maintained upon thawing of cryopreserved samples (Figures 2 and 3). Other imaging modalities may lack the ability to be cryopreserved and require labeling of the cellular population after thawing due to the nature of the imaging agent (i.e., half-life) or concerns regarding the extent of cellular labeling [28].

Incorporating a cellular imaging agent to the therapeutic process allows PK data to be achieved through* in vivo* imaging of cellular therapies. Imaging enables the examination of a therapy's ability to reach its target (such as a solid tumor). Of equal importance, such methodology could account for potential off target delivery. In the selection of route of administration for cellular delivery, comparing various routes could help drive therapeutic outcome if one route demonstrates improved targeting. Furthermore, since therapeutic response may take 90 to 120 days, the possibility of using an imaging agent as a biomarker for efficacy when considering cancer treatment also holds great promise. Imaging also allows for the detection of cells failing to home/migrate; should a cellular therapy not reach the site of disease, readministration of the cellular therapy of alternative therapies could also be considered. Our goal by addressing the scalability of cellular labeling is that the technology can enable faster and more effective examination of cellular PK in man.

Imaging in clinical trials has the potential to strengthen safety and homing data. With one trial using 19F imaging completed [19] and another trial set to recruit (clinicaltrials.gov, NCT02035085) there are still steps being made in the implementation of this technology. Through the data developed in this manuscript, the concerns over clinical scale-up and incorporation of cellular labeling in clinical scale T-cell immunotherapy manufacturing have been addressed. As engineered T-cells are developed to target solid tumors, the ability to image with an agent that can transition from preclinical to clinical studies can help develop the pharmacokinetic data to necessary to accelerate the therapeutic path forward.

## Abbreviations

CAR: Chimeric Antigen Receptor
TCR: T-Cell Receptor
ALL: Acute Lymphoblastic Leukemia
PK: Pharmacokinetic
19F: Fluorine 19
NK: Natural Killer
MRI: Magnetic Resonance Imaging
PET: Positron emission tomography
SPECT: Single photon emission computed tomography
CS: Cell Sense.
